# Supplementary material for: Disturbance has lasting effects on functional traits and diversity of grassland plant communities
Source: PeerJ. 2022 Mar 25;10:e13179. doi: 10.7717/peerj.13179 (PMC8958970; doi:10.7717/peerj.13179)
Supplement: Supplemental Information 1 [file peerj-10-13179-s001.docx]

|  | **2018 Undisturbed** | **2020 Undisturbed** | **2020 Disturbed** |
| --- | --- | --- | --- |
|  | *Achillea millefolium* | *Achillea millefolium* | *Achillea millefolium* |
|  | *Agropyron dasystachyum* | *Agropyron dasystachyum* | *Agropyron dasystachyum* |
|  | *Agrostis scabra* | *Agrostis scabra* | *Agrostis scabra* |
|  | *Anemone cylindrica* |  |  |
|  | *Antennaria neglecta* | *Antennaria neglecta* |  |
|  |  |  | *Androsace septentrionalis* |
|  | *Antennaria parvifolia* | *Antennaria parvifolia* | *Antennaria parvifolia* |
|  | *Artemisia frigida* | *Artemisia frigida* | *Artemisia frigida* |
|  | *Artemisia ludoviciana* | *Artemisia ludoviciana* | *Artemisia ludoviciana* |
|  | *Astragalus agrestis* | *Astragalus agrestis* | *Astragalus agrestis* |
|  | *Astragalus flexuosus* | *Astragalus flexuosus* | *Astragalus flexuosus* |
|  |  | *Boecherea divaricarpa* | *Boecherea divaricarpa* |
|  | *Bouteloua gracilis* | *Bouteloua gracilis* | *Bouteloua gracilis* |
|  | *Bromus inermis* | *Bromus inermis* | *Bromus inermis* |
|  | *Campanula rotundifolia* | *Campanula rotundifolia* | *Campanula rotundifolia* |
|  | *Carex*spp. | *Carex*spp. | *Carex*spp. |
|  | *Cerastium arvense* | *Cerastium arvense* | *Cerastium arvense* |
|  | *Chenopodium album* |  | *Chenopodium album* |
|  |  | *Cirsium vulgare* | *Cirsium vulgare* |
|  |  |  | *Cirsium arvense* |
|  |  | *Collomia linearis* | *Collomia linearis* |
|  | *Commandra umbellata* | *Commandra umbellata* |  |
|  | *Danthonia intermedia* |  |  |
|  | *Draba nemorosa* |  |  |
|  | *Drymocallis arguta* |  |  |
|  | *Eleagnus commutata* |  | *Eleagnus commutata* |
|  | *Elymus trachycaulus* | *Elymus trachycaulus* | *Elymus trachycaulus* |
|  | *Erigeron caespitosus* | *Erigeron caespitosus* | *Erigeron caespitosus* |
|  |  | *Erigeron glabellus* | *Erigeron glabellus* |
|  | *Erysimum inconspicuum* |  | *Erysimum inconspicuum* |
|  | *Fallopia convolvulus* | *Fallopia convolvulus* |  |
|  | *Festuca halli* | *Festuca halli* | *Festuca halli* |
|  | *Fragaria virginiana* | *Fragaria virginiana* |  |
|  | *Gaillardia aristata* | *Gaillardia aristata* |  |
|  | *Galium boreale* | *Galium boreale* | *Galium boreale* |
|  | *Gentianella amarella* | *Gentianella amarella* | *Gentianella amarella* |
|  |  | *Geum aleppicum* |  |
|  | *Geum triflorum* | *Geum triflorum* | *Geum triflorum* |
|  | *Helictochloa hookeri* |  |  |
|  | *Hesperostipa curtiseta* | *Hesperostipa curtiseta* | *Hesperostipa curtiseta* |
|  | *Heuchera richardsonii* |  |  |
|  | *Koeleria macrantha* | *Koeleria macrantha* |  |
|  |  |  | *Mulgedium pulchellum* |
|  | *Nassella viridula* | *Nassella viridula* | *Nassella viridula* |
|  | *Orthocarpus luteus* | *Orthocarpus luteus* | *Orthocarpus luteus* |
|  | *Oxytropis campestris* | *Oxytropis campestris* | *Oxytropis campestris* |
|  | *Pascopyrum smithii* | *Pascopyrum smithii* | *Pascopyrum smithii* |
|  | *Penstemon gracilis* | *Penstemon gracilis* | *Penstemon gracilis* |
|  | *Poa interior* | *Poa interior* |  |
|  | *Poa pratensis* | *Poa pratensis* | *Poa pratensis* |
|  | *Potentilla concinna* | *Potentilla concinna* | *Potentilla concinna* |
|  | *Potentilla hippiana* | *Potentilla hippiana* |  |
|  | *Pulsatilla nuttalliana* | *Pulsatilla nuttalliana* | *Pulsatilla nuttalliana* |
|  | *Rosa arkansana* | *Rosa arkansana* | *Rosa arkansana* |
|  |  | *Rubus occidentalis* | *Rubus occidentalis* |
|  |  | *Sisyrinchium montanum* | *Sisyrinchium montanum* |
|  | *Solidago missouriensis* | *Solidago missouriensis* | *Solidago missouriensis* |
|  |  | *Sonchus arvensis* | *Sonchus arvensis* |
|  | *Symphoricarpos occidentalis* |  | *Symphoricarpos occidentalis* |
|  | *Symphyotrichum falcatum* | *Symphyotrichum falcatum* | *Symphyotrichum falcatum* |
|  | *Symphyotrichum laeve* | *Symphyotrichum laeve* | *Symphyotrichum laeve* |
|  | *Taraxacum officinale* | *Taraxacum officinale* | *Taraxacum officinale* |
|  |  |  | *Thalictrum venulosum* |
|  | *Thermopsis rhombifolia* | *Thermopsis rhombifolia* | *Thermopsis rhombifolia* |
|  | *Tragopogon dubius* |  | *Tragopogon dubius* |
|  |  |  | Unidentified mustard |
|  |  |  | Unidentified weed |
|  | *Vicia americana* | *Vicia americana* | *Vicia americana* |
|  | *Viola adunca* | *Viola adunca* |  |
| **Total Richness** | **55** | **52** | **53** |
