# Supplementary material for: Disturbance has lasting effects on functional traits and diversity of grassland plant communities
Source: PeerJ. 2022 Mar 25;10:e13179. doi: 10.7717/peerj.13179 (PMC8958970; doi:10.7717/peerj.13179)
Supplement: Supplemental Information 2 — Community weighted means for specific leaf area (SLA), height, shoot percent nitrogen (shoot N %), root percent nitrogen (root N %), and specific root length (SRL) were used as response variables in separate models. Plot pair (i.e., disturbed and undisturbed plot location) was included as a random effect in linear mixed models. Plot type (disturbed or undisturbed) was the fixed effect in linear and linear mixed models. SE stands for standard error. Bold type indicates significant results. [file peerj-10-13179-s002.docx]

|  | Linear Mixed Model Outputs | | | Linear Model Outputs | | |
| --- | --- | --- | --- | --- | --- | --- |
|  | Disturbed plot mean ± SE | Undisturbed plot mean ± SE | Linear Mixed Model  *p*-value | Disturbed plot mean ± SE | Undisturbed plot mean ± SE | Linear Model *p*-value |
| Maximum height (cm) | 35.78 (± 1.05) | 36.30 (± 1.16) | 0.66 | 35.78 (± 1.05) | 36.30 (± 1.48) | 0.73 |
| Specific leaf area (cm^2^/g) | 139.96 (± 2.99) | 119.34 (± 2.99) | **2.64 x 10^-5^** | 139.96 (± 2.99) | 119.34 (± 4.23) | **7.2 x 10^-5^** |
| Specific root length (cm/g) | 5612.82 (± 448.19) | 4550.30 (± 570.50) | 0.09 | 5612.82 (± 448.2) | 4550.3 (± 633.8) | 0.11 |
| Leaf nitrogen (%) | 1.92 (± 0.04) | 1.74 (± 0.06) | **0.01** | 1.92 (± 0.04) | 1.74 (± 0.06) | **0.01** |
| Root nitrogen (%) | 1.05 (± 0.03) | 0.95 (± 0.04) | **0.02** | 1.05 (± 0.03) | 0.95 (± 0.04) | **0.02** |
