# Supplementary figures and images for: Disturbance has lasting effects on functional traits and diversity of grassland plant communities
Source: PeerJ. 2022 Mar 25;10:e13179. doi: 10.7717/peerj.13179 (PMC8958970; doi:10.7717/peerj.13179)

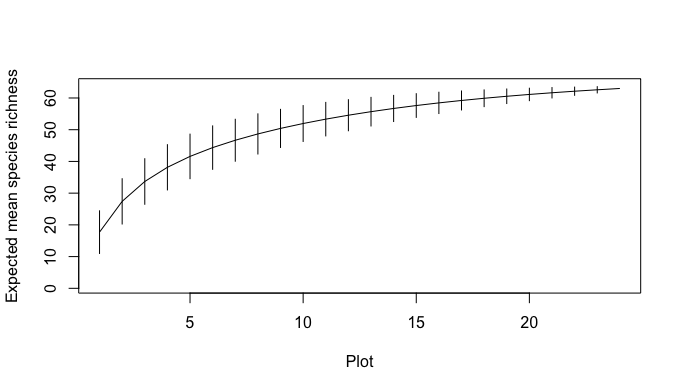

Supplement: Supplemental Information 4 — Species richness data from disturbed and undisturbed plots was used to create the curve. [file peerj-10-13179-s004.png]
